# Impact of Data Quality on Deep Learning Prediction of Spatial Transcriptomics from Histology Images

**DOI:** 10.1101/2025.09.04.674228

**Published:** 2025-09-09

**Authors:** Caleb Hallinan, Calixto-Hope G. Lucas, Jean Fan

**Affiliations:** 1 Center for Computational Biology, Whiting School of Engineering, Johns Hopkins University, Baltimore, MD 21211, USA; 2 Department of Biomedical Engineering, Johns Hopkins University, Baltimore, MD 21211, USA; 3 Department of Pathology, Johns Hopkins University, Baltimore, MD 21287, USA; 4 Department of Neurosurgery, Johns Hopkins University, Baltimore, MD 21287, USA

**Keywords:** spatial transcriptomics, gene prediction, digital pathology, benchmark, machine learning, deep learning, data-centric AI

## Abstract

Spatial transcriptomics technologies enable high-throughput quantification of gene expression at specific locations across tissue sections, facilitating insights into the spatial organization of biological processes. However, high costs associated with these technologies have motivated the development of deep learning methods to predict spatial gene expression from inexpensive hematoxylin and eosin-stained histology images. While most efforts have focused on modifying model architectures to boost predictive performance, the influence of training data quality remains largely unexplored. Here, we investigate how variation in molecular and image data quality stemming from differences in imaging (Xenium) versus sequencing (Visium) spatial transcriptomics technologies impact deep learning-based gene expression prediction from histology images. To delineate the aspects of data quality that impact predictive performance, we conducted *in silico* ablation experiments, which showed that increased sparsity and noise in molecular data degraded predictive performance, while *in silico* rescue experiments via imputation provided only limited improvements that failed to generalize beyond the test set. Likewise, reduced image resolution can degrade predictive performance and further impacts model interpretability. Overall, our results underscore how improving data quality offers an orthogonal strategy to tuning model architecture in enhancing predictive modeling using spatial transcriptomics and emphasize the need for careful consideration of technological limitations that directly impact data quality when developing predictive methodologies.

## Introduction

Spatial transcriptomics (ST) technologies enable the measurement of gene expression with spatial context across tissue sections with varying molecular resolution and throughput. These spatial molecular measurements are often accompanied by a hematoxylin and eosin (H&E)-stained image of the tissue section used for the ST assay. Although ST experiments can cost several thousand dollars per sample, H&E-stained images are relatively inexpensive and routine in clinical and research histopathology. By leveraging such matched H&E-stained images and gene expression data, machine learning models can be trained to predict molecular information directly from histology images. This greatly extends the utility of both archived and newly generated histology images for molecular inference, potentially bypassing the need for expensive ST technologies.

The quality of molecular data can vary widely across ST technologies, reflecting the unique technical constraints inherent to each technology. Imaging-based technologies (for example, STARmap [1], MERFISH [2], and 10x Xenium) provide sub-cellular resolution and typically higher sensitivity but are currently limited to smaller gene panels and can suffer from optical signal overlap, off-target probe-binding issues, and cell-segmentation errors [3–5]. In contrast, sequencing-based technologies (for example, Slide-seqV2 [6], DBiT-seq [7], and 10x Visium) provide transcriptome-wide coverage but at generally lower spatial resolution and can be prone to lateral diffusion between spots, problematic spots, as well as PCR amplification biases and drop-outs that yield sparse, noisy counts [8–11]. In addition to these ST technology-specific limitations impacting the molecular data quality, the quality of histology images can also vary through differences in staining protocols, scanner optics, image compression, and other factors that alter color balance, contrast, and effective pixel size across datasets [12, 13]. Although multiple sources contribute to variation in data quality, their collective impact on the performance and interpretability of downstream predictive models remains largely unexplored.

A growing body of work has applied deep learning models, varying in terms of the convolutional, graph, or transformer-based architectures used, to predict gene expression from histology images using ST data for training [14, 15]. At first, these approaches were almost exclusively trained and evaluated on data from sequencing-based technologies such as Visium where predictive performance, as measured by the Pearson correlation coefficient between predicted and observed gene expression, often averaged around 0.3. More recent studies have begun training and evaluating models using data from imaging-based technologies such as Xenium, reporting a higher predictive performance [16, 17]. However, prediction performance remains highly variable across datasets, tissue types, and models, highlighting the need for further investigation into factors that impact prediction performance [18].

In light of this, our study assesses the impact of training data quality on the performance of gene expression prediction from histology images using deep learning models. To this end, we utilize two previously published breast cancer datasets from serial tissue sections assayed by a sequencing-based technology (Visium) and an imaging-based technology (Xenium) with paired H&E-stained images of the same tissue section [19]. We find that when using Xenium data as the input training data compared to when using Visium data, the average prediction performance across genes increased. To determine what specific data quality factors in the input training data may contribute to the observed prediction performance differences between technologies, we further perform *in silico* ablation experiments to reduce data quality by introducing expression sparsity and noise and decreasing image resolution on the better performing Xenium data to recapitulate poor performance when trained with the worse performing Visium dataset. We further perform *in silico* rescue experiments in an attempt to enhance molecular data quality of the Visium dataset using gene expression imputation and demonstrate that such augmentation leads to overfitting and poor generalizability. Overall, our results underscore the critical role of training data quality in spatial gene expression prediction and demonstrates that training on higher-quality data can improve prediction performance.

## Results

### Data quality benchmarking on paired Visium and Xenium spatial transcriptomics datasets

**Figure 1:**
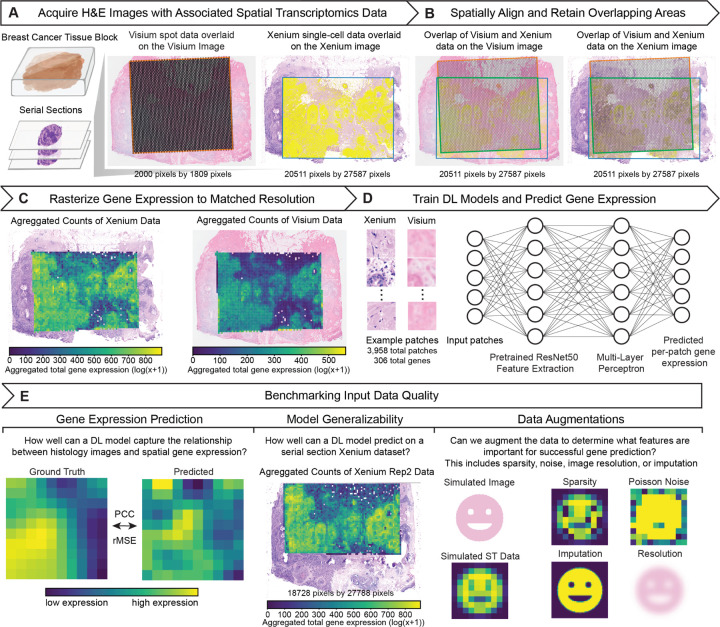
Overview of the data-quality benchmarking pipeline. **A.** Acquisition of paired breast cancer spatial transcriptomics datasets and histology images from 10x Visium and Xenium. **B.** Co-registration of Visium and Xenium histology slides into a common coordinate system. The green box highlights the overlapping region retained between the two technologies. **C.** Rasterization of gene counts onto a uniform grid matched to Visium spot resolution, followed by extraction of the overlapping tissue region. Expression is visualized as patches. **D.** Training of deep learning models to predict per-patch gene expression from histology image patches. **E.** Performance evaluation on held-out replicates, comparison across technologies, and ablation experiments of inputs.

To investigate how ST training data quality affects gene expression prediction from histology images via deep learning, we leveraged serial section breast cancer datasets generated by Visium CytAssist and Xenium (Figure 1A) [19]. Because a single tissue section cannot undergo two distinct ST assays, serial sections allow us to control for biological variability and focus on technical variability across the different ST technologies. Still, slight differences in the region of the tissues profiled as well as image and molecular data resolution persist. Therefore, to better ensure that differences in gene expression prediction performance can be attributed to the training regime rather than to confounding variation between the training datasets, we standardize as many aspects of the input as possible. To this end, we first aligned the Visium histology image to the Xenium histology image using STalign, bringing both datasets into a common coordinate system (Figure 1B) [20]. This enables the molecular data from either technology to be visualized and utilized with either histology image. We then identified the shared region between the two datasets and rasterized the gene expression to a common spatial resolution (Figure 1C), as demonstrated by Aihara et al. [21]. By minimizing such sample-specific differences, this preprocessing enables controlled comparisons across all combinations of molecular and imaging data. Using this setup, we extracted histology image patches that correspond to the rasterized molecular data and trained identical deep learning models composed of a pretrained ResNet50 feature extractor, a four-layer multilayer perceptron and a final linear output layer to predict gene expression at the patch level for the 306 genes common to both ST assays (Figure 1D). We evaluated model prediction performance by computing Pearson correlation coefficients (PCC) and normalized root mean square error (rMSE) between true and predicted patch-level gene expression on a held-out test set (15% of patches) and, in some cases, on an independent serial section Xenium replicate dataset (Rep2) to further assess generalizability. To determine which factors of data quality influence prediction performance and interpretability, we performed a series of data augmentations including increasing expression sparsity and noise, applying Gaussian blur to simulate lower-resolution images, as well as gene expression imputation in an attempt to remove sparsity and noise, and then reassessed prediction performance as well as Grad-CAM saliency (Figure 1E).

**Figure 2:**
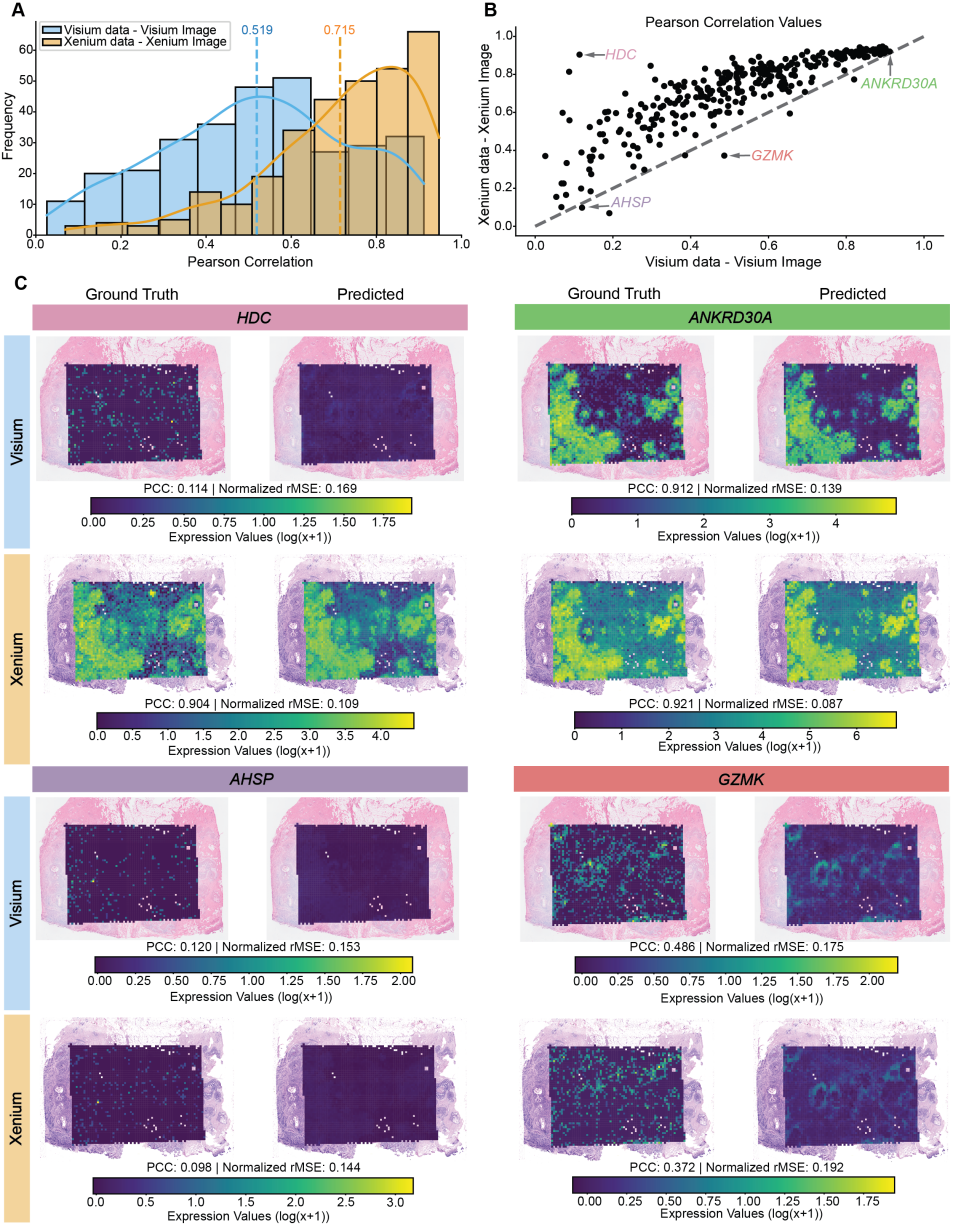
Spatial gene expression prediction comparison using Visium vs. Xenium data. **A.** Histogram showing the distribution of Pearson correlation coefficients for gene expression predictions using Visium and Xenium data. The dotted vertical line denotes the mean PCC, and the solid curved line traces the density estimate. Results are computed on the held-out test set and represent the average performance across five independently trained models. **B.** Scatterplot comparing the Pearson correlation coefficients of predictions from Visium and Xenium data. The gray dotted line denotes x=y, and select genes corresponding to (C) labeled. **C.** Representative examples of ground truth and predicted gene expression for *HDC*, *ANKRD30A*, *AHSP*, and *GZMK* in both the Visium and Xenium datasets. Predicted gene expressions are visualized for the full dataset, while the performance metrics (PCC and normalized rMSE) are computed from the held-out test set only.

Applying this approach, we directly compared the predictive performance of our deep learning model when trained on each technologies’ associated histology image and corresponding molecular data (Figure 2). For every gene, we computed the PCC between the ground-truth expression values and model predictions on the held-out test set, using five independently trained models to account for training variability. We found that the PCC distribution across genes is notably shifted toward higher values, indicative of better prediction performance, when trained on Xenium data compared to Visium data (mean PCC = 0.715 for Xenium vs. 0.519 for Visium; Figure 2A), representing an increased average prediction performance across genes of approximately 38%. Likewise, in the gene-by-gene scatterplot (Figure 2B), almost all points lie above the diagonal indicating that prediction performance when training on Xenium data was consistently higher than with Visium data for almost all genes. Evaluating the complete distribution of ground-truth and predicted expression values for held-out test set patches reveals that the PCC is not driven by outliers (Supplementary Figure 1). To further ensure this trend holds across metrics, we also examined the range-normalized root mean square error (rMSE) and found that gene expression predictions compared to the ground-truth expression values exhibited higher normalized rMSE, indicative of worse performance, when trained on Visium data compared to Xenium data (Supplementary Figure 2). Plots of observed versus predicted expression for four representative genes (*HDC*, *ANKRD30A*, *AHSP*, *GZMK*) highlight varying performance outcomes: Xenium-trained models outperform Visium for *HDC*, both models perform similarly well for *ANKRD30A* and poorly for *AHSP*, while Visium-trained models slightly outperforms Xenium for *GZMK* (Figure 2C). Overall, these results indicate that models trained on Xenium’s paired molecular data and histology image achieve stronger gene-level correlations between predicted and ground-truth gene expression magnitudes than those trained on the Visium molecular data and its corresponding histology image. Whether this advantage arises primarily from differences in molecular data quality or from image quality or both remains to be determined in our subsequent analyses.

### Molecular data quality can impact spatial gene expression prediction performance

**Figure 3:**
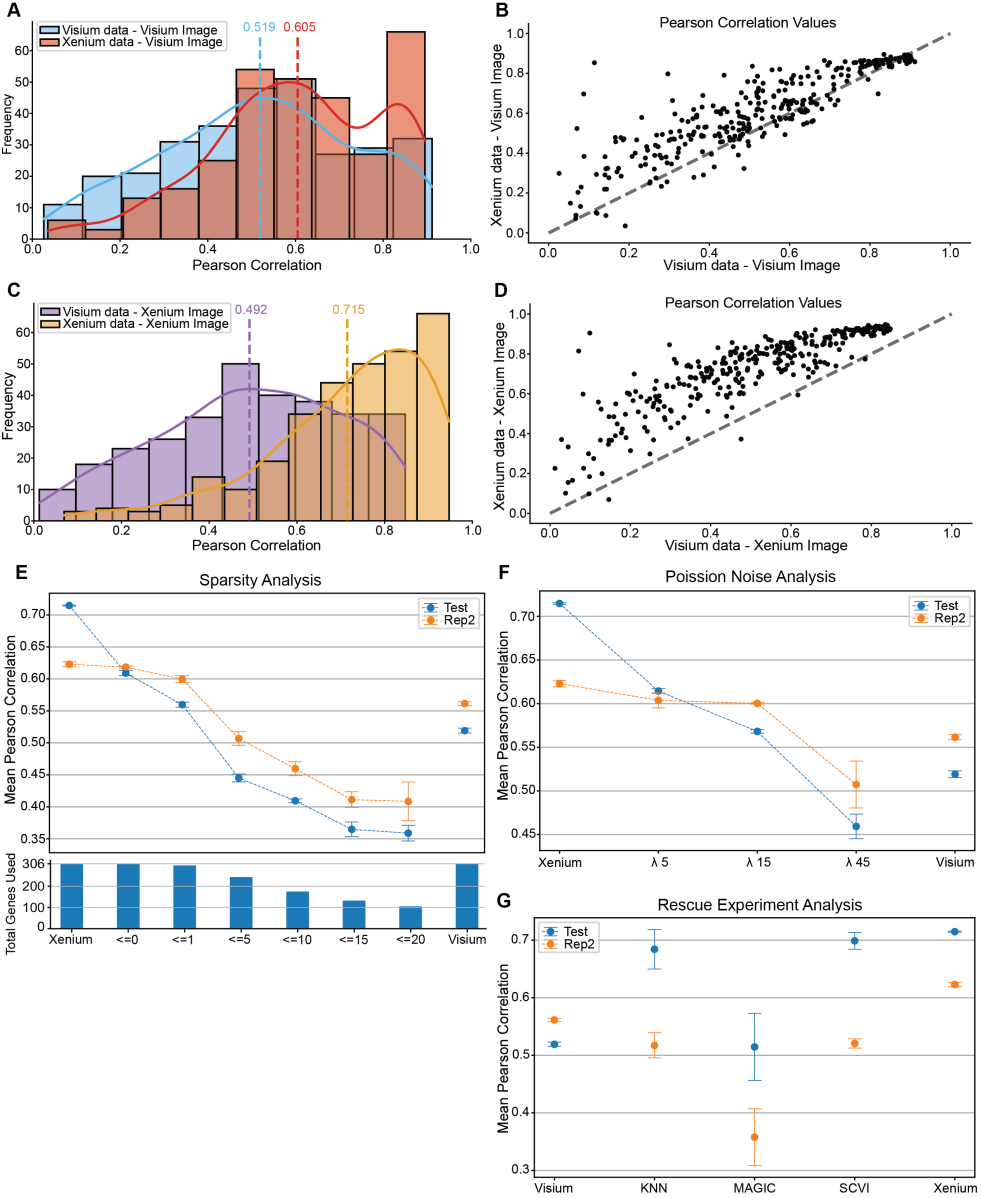
Impact of molecular data quality on spatial gene expression prediction. **A.** Histogram of Pearson correlation coefficients for gene expression predictions using Visium and Xenium data with the Visium image. The dotted vertical line denotes the mean PCC, and the solid curved line traces the density estimate. **B.** Scatterplot comparing PCC values from Visium and Xenium data with the Visium image on the test set, averaged across five models. The gray dotted line denotes x=y. **C.** Histogram of PCC values for predictions using Visium and Xenium data with the Xenium image. The dotted vertical line denotes the mean PCC, and the solid curved line traces the density estimate. **D.** Scatterplot comparing PCC values from Visium and Xenium data with the Xenium image on the test set, averaged across five models. The gray dotted line denotes x=y. **E.** Scatterplot comparing PCC values between Xenium, an increasing amount of sparsity in the Xenium dataset, and the Visium results on the test and replicate 2 Xenium data. The dotted line indicates the dataset used, and error bars represent the standard error across five runs. The histogram below denotes the total number of genes used to calculate the mean PCC. **F.** Scatterplot comparing PCC values between Xenium, an increasing amount of Poisson noise in the Xenium dataset, and the Visium results on the test and replicate 2 Xenium data. The dotted line indicates the dataset used, and error bars represent the standard error across five runs. **G.** Scatterplot comparing PCC values between Visium, various imputation methods on the Visium dataset, and the Xenium results on the test and replicate 2 Xenium data. The dotted line indicates the dataset used, and error bars represent the standard error across five runs.

To disentangle the impact of molecular data quality from image data quality, we next held the image data constant while swapping molecular data between technologies. We leveraged the high-resolution histology image from the Visium dataset and trained two identical models: one on Visium molecular data, the other on Xenium molecular data (Figure 3A–B). Under these conditions, Xenium molecular data outperformed Visium molecular data (mean PCC = 0.605 vs. 0.519; Figure 3A), and most genes exhibited higher PCCs when trained with the Xenium molecular data (Figure 3B). We next repeated this with the whole-slide image (WSI) from the Xenium dataset. Again, models trained using Xenium molecular data outperformed Visium molecular data (mean PCC = 0.715 for Xenium vs. 0.492 for Visium; Figure 3C–D). These trends further held for normalized rMSE (Supplementary Figure 3). This demonstrates that, irrespective of the image data, the molecular data used for training has an effect on gene expression prediction performance.

To pinpoint what molecular data quality factors may drive these performance differences, we next performed a series of *in silico* ablation experiments in which we systematically decrease molecular data quality and evaluate its impact on prediction performance. First, we examined expression sparsity, defined here as the proportion of zero counts across patches for each gene. Consistent with previous research, we note that sparsity is more pronounced in the molecular data from Visium than Xenium (Supplementary Figure 4). Having confirmed Xenium data’s better performance, we increased sparsity in the patch-resolution Xenium gene expression count matrix to match the sparsity in the Visium gene expression count matrix. To do this, we compared corresponding patches from the Visium and Xenium datasets, and wherever the Visium count matrix exhibited a value of ≤ 0, we set the corresponding patch in the patch-resolution Xenium count matrix value to 0. We repeated this process for Visium expression thresholds of ≤ 1, ≤ 5, ≤ 10, ≤ 15, and ≤ 20. As sparsity increased, mean PCC on the held-out test set steadily declined (Figure 3E). To ensure this effect was not an artifact of the held-out test data and to access model generalization on a new dataset, we repeated the evaluation on an independent Xenium serial section (Rep2). Here too, we observed a similar downward trend in PCC as sparsity increased (Figure 3E). Notably, training with Xenium molecular data with increased sparsity using a ≤ 1 threshold produced comparable performance to when training with the Visium molecular data. Next, we examined expression noise, defined as random fluctuations in gene counts around their true values. To simulate greater expression noise, we added Poisson distributed noise into the patch-resolution Xenium count matrix across a series of rate parameters (*λ*). As we increased *λ* to simulate more expression noise, mean PCC on the held-out test set steadily declined (Figure 3F). We again repeated the evaluation on the independent Xenium serial section (Rep2) to confirm this trend. Overall, these results implicate molecular data sparsity and noise as potential drivers of decreased prediction performance.

Finally, given that increased sparsity and noise can degrade prediction performance, we asked whether we could rescue the Visium molecular data via gene imputation. Gene imputation refers to techniques that infer and fill in missing or zeroed expression values by borrowing information from similar cells or genes across the dataset or smoothing expression profiles to mitigate sparsity and noise issues. We applied three representative imputation methods: KNN [22], MAGIC [23], and SCVI [24] to the Visium molecular data, retrained our models, and evaluated PCC on the test set and on the independent replicate (Figure 3G). Applying these imputation methods boosted the PCC when evaluated on the held-out test set. However, when we repeated evaluation on the independent Xenium replicate, applying these imputation methods led to a consistent decrease in the PCC. This suggests that while imputation can mask sparsity and noise to aid model training, it may introduce biases that limit robustness and generalizability to new samples.

### Image data quality can impact spatial gene expression prediction performance

**Figure 4:**
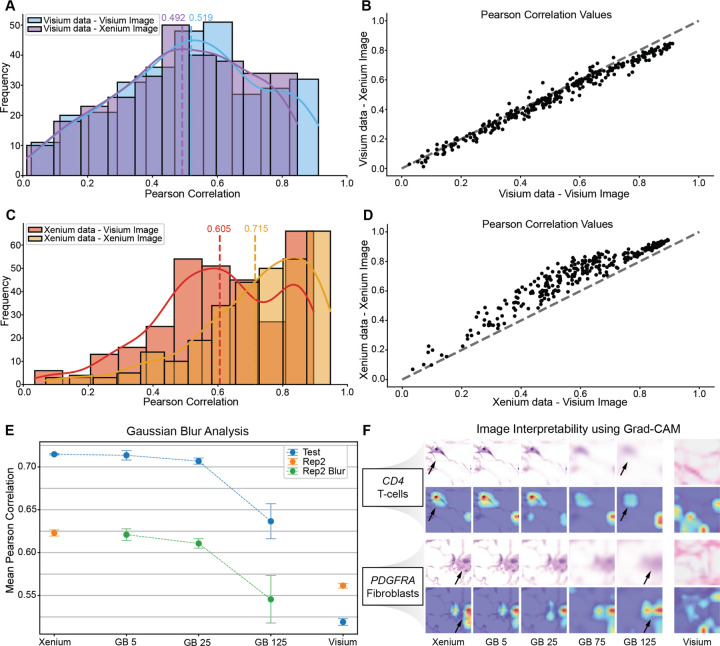
Impact of imaging data quality on spatial gene expression prediction. **A.** Histogram showing the distribution of Pearson correlation coefficients for gene expression predictions using Visium data with the Visium and Xenium images. The dotted vertical line denotes the mean PCC, and the solid curved line traces the density estimate. Results are computed on the test set and represent the average performance across five independently trained models. **B.** Scatterplot comparing the Pearson correlation coefficients of predictions from Visium data with the Visium and Xenium images, based on the test set and averaged over five models. The gray dotted line denotes x=y. **C.** Histogram showing the distribution of Pearson correlation coefficients for gene expression predictions using the Xenium data with the Visium and Xenium image. The dotted vertical line denotes the mean PCC, and the solid curved line traces the density estimate. Results are computed on the test set and represent the average performance across five independently trained models. **D.** Scatterplot comparing the Pearson correlation coefficients of predictions from Xenium data with the Visium and Xenium image, based on the test set and averaged over five models. The gray dotted line denotes x=y. **E.** Scatterplot of mean Pearson correlation coefficients on both the test set and the Replicate 2 Xenium section, comparing the Xenium, Xenium images with increasing Gaussian blur, and Visium results (all applied with the same blur levels). The dotted line indicates the dataset used, and error bars represent the standard error of the mean across five independent model runs. **F.** Grad-CAM heatmaps for two select genes: *CD4* (T-cell marker) and *PDGFRA* (fibroblast marker).

To isolate the effect of image data quality from molecular data quality, we held the molecular input constant and swapped histology images between technologies. First, using Visium molecular data, we trained models on both the Visium high-resolution image (2000px by 1809px)and the higher resolution Xenium WSI (20511px by 27587px). The resulting PCC distributions were comparable (mean PCC = 0.519 with Visium image vs. 0.492 with Xenium image; Figure 4A). Gene-level correlations remained along the diagonal (Figure 4B), and normalized rMSE values were similarly low (Supplementary Figure 5A), indicating that image resolution had little effect on prediction performance. Next, using Xenium molecular data, we repeated the experiment and observed a larger performance gap (mean PCC = 0.605 with Visium image vs. 0.715 with Xenium image; Figure 4C). Gene-by-gene scatterplots (Figure 4D) and normalized rMSE comparisons (Supplementary Figure 5B) demonstrate consistent trends. As such, training on higher resolution histology images can improve prediction performance depending on the quality of the corresponding molecular data.

To pinpoint what imaging data quality factors may drive these performance differences, we simulated lower-resolution images by applying Gaussian blur with kernels of size 5, 25, and 125 to the Xenium histology images. Under matched conditions, training and testing on equally blurred images, PCC slowly degraded with decreasing simulated image resolution (Figure 4E). These results suggest that image resolution has a measurable impact on gene expression prediction performance, with progressively blurred images leading to decreased prediction performance.

Finally, beyond the impact of image resolution on gene expression prediction performance, we sought to assess its impact on model interpretability. To this end, we employed Grad-CAM [25], which projects prediction gradients onto convolutional feature maps to generate heatmaps that highlight the image regions most influential for a given output. This technique is especially useful because it can reveal whether the model focuses on potentially biologically meaningful structures, such as cells or nuclei consistent with what histopathologists may focus on, rather than background artifacts. We applied Grad-CAM to predictions for select well-predicted genes with high PCCs including *CD4* (PCC = 0.79), a T-cell marker, and *PDGFRA* (PCC = 0.76), a fibroblast marker (Figure 4F). On high-resolution images, the resulting heatmaps highlighted cellular and nuclear regions. As we introduced increasing levels of blur to simulate reduced resolution, the heatmaps became diffuse and lost alignment with these structural landmarks. These results demonstrate that lower image resolution may not only decrease gene expression prediction performance but also reduce reliable interpretation of the tissue features that drive model predictions.

## Discussion

In this study, we evaluated how data quality impacts deep learning–based gene expression prediction from histology images. We leveraged molecular data paired with H&E-stained images from two different ST technologies, one imaging-based (Xenium) and one sequencing-based (Visium), from serial sections of the same breast cancer tissue thereby representing ST data with varying data quality features. We trained identical deep learning models to predict patch-level gene expression and observed that training on Xenium data can achieve quantifiably better gene expression prediction performance than training on Visium data, with an average prediction performance across genes increase of approximately 38%. To isolate the impact of molecular data quality, we held the histology image constant and found that Xenium molecular data outperformed Visium molecular data regardless of which image was used. In molecular ablation experiments, adding noise and sparsity to Xenium molecular data steadily degraded prediction performance. Conversely, in molecular rescue experiments, imputing Visium data boosted performance on the held-out test set but failed to generalize to an independent Xenium replicate. Likewise, to isolate the impact of image data quality, we held the molecular data constant and found Visium molecular data yielded similar prediction performance on both the Visium and Xenium images, whereas Xenium molecular data benefited from the higher-resolution Xenium image. Simulating lower-resolution imaging by applying Gaussian blur to the Xenium image reduced predictive performance and obscured interpretability via Grad-CAM feature maps. Overall, these findings underscore the impact of both molecular and imaging data quality in deep learning–based gene expression prediction from histology images. Further, our study presents quantifiable metrics of molecular and imaging data quality in terms of their sparsity, noise, and resolution that can be considered in the curation of training data from ST technologies for developing gene expression prediction methodologies.

Despite these insights, our study has several limitations. Although our comparative analysis demonstrated that training on Visium data resulted in lower gene expression prediction performance than training on Xenium data, potentially attributable to greater sparsity, noise, and other data quality issues in Visium, Xenium data also exhibits its own limitations in data quality. For example, previous research has demonstrated that imaging-based technologies like Xenium can suffer from off-target probe hybridization, thereby obscuring the target gene’s expression with the off-target gene’s expression [3]. This may result in an increase in the observed target gene’s expression magnitude. Because previous studies have observed that genes with higher mean expression are generally easier to predict, these off-target artifacts could artificially inflate the prediction performance from training on Xenium molecular data [16]. We therefore used the Off-Target Probe Tracker (OPT) with a pad-length of 10 to identify 16 of our 306 genes with potential protein-coding off-target genes [3]. Removing these genes and retraining our models confirmed that they do not drive our observed trends or affect our conclusions (Supplementary Figure 6). Nevertheless, comparing predictions of gene expression using datasets from different ST technologies inherently assumes these different ST technologies are measuring the same underlying genes, which can be obscured by off-target probe binding. Hence, future work is still needed to quantify off-target rates as well as develop robust correction strategies to ensure fair, accurate comparisons across ST technologies.

Similarly for image data quality, beyond image resolution, other features in H&E staining and slide preparation can impact the performance of deep learning models. In particular, H&E staining for Xenium is acquired after the *in situ* gene expression profiling is complete, whereas H&E staining for Visium is acquired before transcriptomic capture. How such protocol variation impacts the quality and generalizability of the H&E-stained images for such predictive modeling purposes remains unclear. Beyond protocol variation, differences in hematoxylin versus eosin concentration, incubation time, and scanner settings can alter color balance, contrast, and texture cues that deep learning models rely on. Common slide artifacts such as air bubbles trapped under the coverslip, uneven tissue folds, tears or wrinkles in the section, dust or debris on the slide and more can further create spurious patterns. Previous studies have shown that these inconsistencies may cause models to learn batch or slide specific artifacts rather than relevant tissue or cellular morphological features [26–29]. As such, we anticipate such common slide artifacts that decrease the quality of the histology image would also impact prediction performance. Likewise, previous studies have sought to address these issues by applying stain-normalization algorithms, color augmentation pipelines, and domain-adaptive foundation models to standardize image appearance across batches and platforms [12, 17, 30, 31]. Future work is needed to evaluate how much such image artifacts impact prediction performance and whether such previously developed strategies are sufficient to rescue poor quality images without introducing biases that limit generalizability. In cases where poor image quality substantially limits predictive performance, re-scanning slides with optimized scanner settings and higher objective lenses could be a practical approach to obtain higher-quality histology images for model training.

Further, our evaluations have been limited to two ST technologies. We focused on 10x Visium and Xenium because, unlike many other current ST technologies, they offer matched H&E-stained images and spatial molecular measurements on the same tissue section. Moreover, the datasets analyzed are derived from serial sections from the same tissue block, thereby allowing us to control for biological variability and focus on technical variability across the different ST technologies. The incorporation of non-serial-section ST datasets from different tissues with distinct data quality features could further enhance our understanding of how such variation affect prediction performance but would also introduce confounders that limit the interpretability of any observed performance differences. Still, the pipeline we present can be generalizable and applied to future serial section datasets from diverse ST technologies as they become available.

Although we found molecular data quality to impact prediction performance, other considerations may come into play in the curation of training data. For example, optimizing for molecular data quality generally comes at the expense of throughput. Therefore, if full-transcriptome gene expression prediction is prioritized, lower-quality datasets with higher throughput may be preferable for training. Cost constraints may also pose challenges to generating high-quality training data. Future work is needed to systematically investigate whether increasing the quantity of training data can compensate for reductions in data quality, and if so, to characterize the rate and extent of such trade-offs.

As with many deep learning applications, interpretability remains a key challenge. Although we can demonstrate quantifiable aspects of image data quality that lead to reduced prediction performance, how or why the models perform worse remains unclear. To this end, we applied Grad-CAM to demonstrate that even modest reductions in image resolution disrupted attention on the cellular and nuclear landmarks (Figure 4F), suggesting that cellular and nuclear landmarks may be important image features in prediction. We therefore hypothesized that predictions of cell-type-specific marker gene expression would direct model attention to corresponding cell-type features, analogous to a pathologist’s assessment. However, we find that even when we switched the predicted gene, attention remained fixed on general cellular and nuclear landmarks in a non-cell-type-specific manner (Supplementary Figure 7). This highlights the persistent challenges in interpreting deep learning interpretability methods. Going forward, the development of more robust explainability techniques, whether advances in Grad-CAM, SHAP [32], Integrated Gradients [33], or brand new approaches will be needed for deep-learning interpretability in this setting, though we anticipate such approaches will need to be paired with sufficiently high-quality images.

Overall, this study underscores the critical role of molecular and image data quality in spatial gene expression prediction. While recent developments have focused on improving modeling approaches, our findings highlight that improvements in training data quality offers an orthogonal strategy to tuning model architecture in enhancing predictive modeling using spatial transcriptomics. Our study further provides a comparative framework for evaluating future ST technologies to characterize aspects of molecular and image data quality to guide this evolving field. Looking forward, we anticipate a more holistic approach that integrates advancements in model architecture with improved generation and curation of training data is likely to yield the greatest performance gains in predictive modeling with spatial transcriptomics. Such a holistic approach will be important for not only improving prediction performance but also enhancing interpretability to ensure reliable and safe future applications in research and clinical settings.

## Methods

### Datasets

Two Xenium datasets (In Situ Replicate 1 and In Situ Replicate 2) along with an adjacent section of a Visium CytAssist dataset of a single breast cancer FFPE tissue block were obtained from the 10x Datasets website for *High-resolution mapping of the breast cancer tumor microenvironment using integrated single cell, spatial and in situ analysis of FFPE tissue* (https://www.10xgenomics.com/products/xenium-in-situ/preview-dataset-human-breast) [19]. Each dataset is accompanied by a histology image. For the Visium CytAssist dataset, both a high-resolution image (2000 pixels by 1809 pixels) and low-resolution image (600 pixels by 543 pixels) are available, with the high-resolution version used in this analysis. The In Situ Replicate 1 Xenium dataset includes a histology image of 20511 by 27587 pixels, while the In Situ Replicate 2 Xenium dataset includes a histology image of 18728 by 27788 pixels. These images are herein referred to as whole-slide images (WSI).

The Visium CytAssist dataset included spatial coordinates of the spots already aligned to its histology image, whereas the two Xenium datasets required aligning segmented cells to their corresponding histology images.

### Restricting to shared tissue regions and shared genes

We used STalign (v1.0.1) to bring the Visium and Xenium data to a common coordinate system by combining affine transformations with diffeomorphic metric mapping [20]. First, we aligned the Visium histology image (source) to the Xenium image (target) using an affine-only registration guided by eight manually selected landmarks. We then applied the learned affine transform to reposition each Visium spot onto the Xenium histology image. This procedure produced co-registered images of the same dimensions, allowing us to overlay Visium spot-level and Xenium single-cell data interchangeably on either histology image. Next, we registered the Xenium transcript coordinates onto their provided histology image using STalign’s full pipeline: rasterize the mapped transcripts at 30*µ*m resolution, an initial affine alignment based on four manually placed landmarks followed by diffeomorphic metric mapping (parameters: *a* = 2500; *epV* = 1; *niter* = 2000; *sigmaA* = 0.11; *sigmaB* = 0.10; *sigmaM* = 0.15; *sigmaP* = 50; *muA* = [1,1,1]; *muB* = [0,0,0]; all other settings default). Finally, we identified the overlapping tissue region between the two technologies (Figure 1B).

Likewise, we restricted our analyses to the 306 genes common to both assays, since Visium provides full-transcriptome coverage while Xenium targets a limited panel.

### Rasterization preprocessing to obtain common patch-resolution training data

After successfully aligning all datasets, histology patches with associated gene expression data were created for input into the deep learning model. For the Visium dataset, each histology patch corresponded to exactly one Visium spot and its associated gene expression values. After aligning the Visium image with the Xenium image, each Visium spot corresponded to a patch size of ∼250×250 pixels. The center coordinates of these Visium patches were then used to generate corresponding patches on the Xenium image. Since the images were aligned, this approach ensured consistency in patch size and location across both datasets. Unlike Visium molecular data, Xenium molecular data corresponds to single cells, meaning each patch contained multiple cells. Therefore, the patch coordinates were used to aggregate (ie. pseudobulk) the Xenium gene expression data into a single spot, analogous to the Visium spots and as done by SEraster [21]. The resulting patch-resolution gene expression counts were then natural log-transformed with a pseudo-count of 1, and patches containing zero gene expression were excluded from both the Visium and Xenium datasets. The resulting histology patches, along with their associated patch-resolution gene expression matrices, were subsequently used as input for the deep learning model. There were a total of 3,958 patches for both datasets.

### Gene imputation, sparsity, and noise data augmentation

Numerous gene imputation methods exist, each representing different functional classes [34]. For this analysis, we selected three methods: KNN [22], MAGIC [23], and SCVI [24].

KNN-smoothing smooths gene expression by identifying nearest neighbors based on partially smoothed and variance-stabilized expression profiles and aggregating their transcript counts. For this analysis, the number of neighbors was set to *k* =50, and *d* =20 top principal components were used to determine the nearest neighbors during each smoothing step. Patch 1587 within the dataset was excluded before applying KNN-smoothing and reintroduced afterward due to insufficient gene counts. The raw UMI counts were used as input, as recommended by the authors.

MAGIC (Markov Affinity-based Graph Imputation of Cells) smooths gene expression by employing a graph-based approach, where nodes represent individual cells and edges encode similarities between them, and diffusing gene expression information across this graph. For this analysis, the number of neighbors was set to *knn* = 50, and the diffusion operator’s power, *t*, was set to ”auto.” All other parameters were kept at their default values. Log-transformed UMI counts with a pseudo-count of 1 were used as input, as recommended by the authors.

ScVI (Single-cell Variational Inference) leverages single-cell RNA-seq data to impute spatial transcriptomic gene expression using a neural network. The neural network was trained using the Chromium 5’ Gene Expression data from another serial section of the breast cancer dataset, along with 80% of the spatial data. All parameters were maintained at their default values, and the model was trained for a maximum of 300 epochs. Genes *TKT*, *SLC39A4*, and *GABARAPL2* were excluded because they were absent from the Chromium 5’ training dataset. Log-transformed UMI counts with a pseudo-count of 1 were used as input.

To introduce sparsity into the Xenium molecular data, Visium patches and their corresponding molecular data are used to impose sparsity on the aligned Xenium patches. Specifically, if a Visium patch contains counts *<*= *t* for gene *x*, the corresponding Xenium patch is set to zero for that gene. This procedure is applied across all patches and repeated at varying count thresholds *t* (0, 5, 10, 15, 20). This approach aims to closely replicate the sparsity observed in Visium molecular data within the Xenium dataset.

To introduce noise into the Xenium molecular data, for each patch we randomly sampled counts from a Poisson distribution with the expected value and variance of (*λ*) and added the sampled values to the original gene counts. The lambda (*λ*) parameter was varied from 5, 15, and 45 to simulate different noise levels.

### Neural network architecture and parameters

For image feature extraction, we used a pre-trained ResNet50 model with the classification layer removed. The input consisted of extracted patches from histology images, sized 250×250×3 which were resized to the standard ResNet50 input size of 224×224×3. The patches were normalized per channel using the mean and standard deviation values from the pre-trained ResNet50 dataset. To enhance model robustness, the patches were subjected to random horizontal, vertical, and 90-degree rotations. The output of the ResNet50 model was the last layer of the model, a feature vector of size 2048, representing extracted image features. Once image features were extracted for each patch, we passed them through a four-layer multilayer perceptron (MLP) to both reduce dimensionality and learn the features most predictive of gene expression. Each MLP layer consisted of a linear transformation, batch normalization, ReLU activation, and 20% dropout. The final output is predicted gene expression for all 306 genes per patch. For training, mean-squared error (MSE) loss was used alongside the Adam optimizer [35], with a learning rate of 0.001 and weight decay of 0.00001. During training, a batch size of 64 was used, and each model was trained for 150 epochs. The datasets were divided into training (75%), validation (10%), and test (15%) sets. All results shown are on the test sets except the plotted predicted gene expression (Figure 2C) which is on the full dataset. Training was performed on an NVIDIA RTX 6000 Ada Generation GPU.

### Deep learning interpretability method

To interpret the regions of the input image that the deep learning model attends to when predicting spatial gene expression, we employed Gradient-weighted Class Activation Mapping (Grad-CAM) [25]. Grad-CAM produces a coarse localization heatmap that highlights the most influential areas of the input image for a given gene prediction. This is achieved by computing the gradient of the target gene score with respect to the feature maps of the final convolutional layer within the ResNet50 architecture. These gradients are then globally averaged to obtain importance weights for each feature map. The weighted feature maps are subsequently combined through a linear summation, and a ReLU activation function is applied to retain only positively contributing regions. The resulting single-channel heatmap is upsampled to match the original input image dimensions, yielding a visual representation of the regions the network considers most relevant for the given gene prediction.

### Overview of metrics used for evaluation

We measured the performance of predicting gene expression with the Pearson Correlation Coefficient (PCC) [36].

$$PCC=\frac{\text{Cov}\left(X_{observed},X_{predicted}\right)}{\sqrt{\text{Var}\left(X_{observed}\right)\text{Var}\left(X_{predicted}\right)}}$$

where

$$\text{Cov}\left(X_{observed},X_{predicted}\right)=\frac{1}{n}\sum_{i=1}^{n}\left(x_{i}-\bar{x}\right)\left(y_{i}-\bar{y}\right),\;\;\text{Var}\left(X_{observed}\right)=\frac{1}{n}\sum_{i=1}^{n}{\left(x_{i}-\bar{x}\right)}^{2},$$

and similarly for $\text{Var}\left(X_{predicted}\right)$. and $X_{predicted}$ are the observed (ground truth) and predicted gene expression, respectively. A higher PCC indicates better predictive performance.

We further measured prediction performance utilizing Normalized Root Mean Squared Error (rMSE), which quantifies the average magnitude of error between observed and predicted values, normalized by the range of observed expression values.

$$\text{normalized}\;\text{rMSE}=\frac{\sqrt{\frac{1}{n}\sum_{i=1}^{n}{\left(y_{i}-{\hat{y}}_{i}\right)}^{2}}}{y_{\max}-y_{\min}}$$

where $y_{i}$ denotes the observed (ground truth) gene expression for observation i, ${\hat{y}}_{i}$ denotes the corresponding model-predicted gene expression, and $y_{\max}$ and $y_{\min}$ are the maximum and minimum observed expression values, respectively. Lower normalized rMSE values indicate better predictive performance, with zero representing a perfect prediction.

## Supplementary Material

Supplement 1
