# Supplementary material for: Impact of Data Quality on Deep Learning Prediction of Spatial Transcriptomics from Histology Images
Source: bioRxiv. 2025 Sep 9:2025.09.04.674228. Preprint. [Version 1] doi: 10.1101/2025.09.04.674228 (PMC12439975; doi:10.1101/2025.09.04.674228)
Supplement: Supplement 1 [file NIHPP2025.09.04.674228v1-supplement-1.pdf]

bioRxiv preprint doi: https://doi.org/10.1101/2025.09.04.674228; this version posted September 9, 2025. The copyright holder for this preprint (which was not certified by peer review) is the author/funder, who has granted bioRxiv a license to display the preprint in perpetuity. It is made available under aCC-BY 4.0 International license.

Supplementary Figures

bioRxiv preprint doi: https://doi.org/10.1101/2025.09.04.674228; this version posted September 9, 2025. The copyright holder for this preprint (which was not certified by peer review) is the author/funder, who has granted bioRxiv a license to display the preprint in perpetuity. It is made available under aCC-BY 4.0 International license.

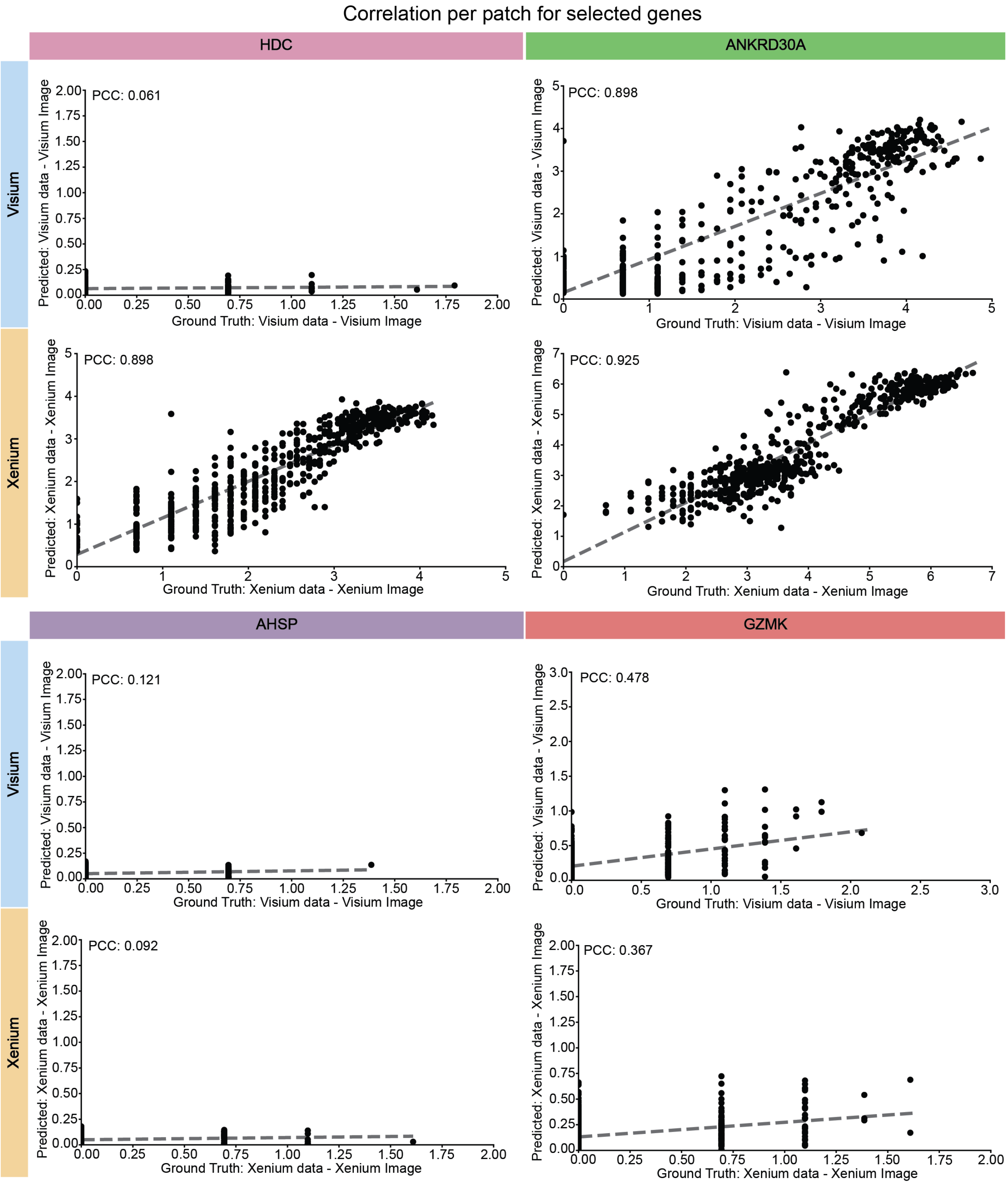

Supplementary Figure 1: Correlation per patch for selected genes. Scatterplots comparing the ground truth held-out test set patches with the predicted expression of patches for *HDC*, *ANKRD30A*, *AHSP*, and *GZMK*. The gray dotted line denotes the linear regression fit. Analyses shown are based on a single seed rather than five independently trained models.

bioRxiv preprint doi: https://doi.org/10.1101/2025.09.04.674228; this version posted September 9, 2025. The copyright holder for this preprint (which was not certified by peer review) is the author/funder, who has granted bioRxiv a license to display the preprint in perpetuity. It is made available under aCC-BY 4.0 International license.

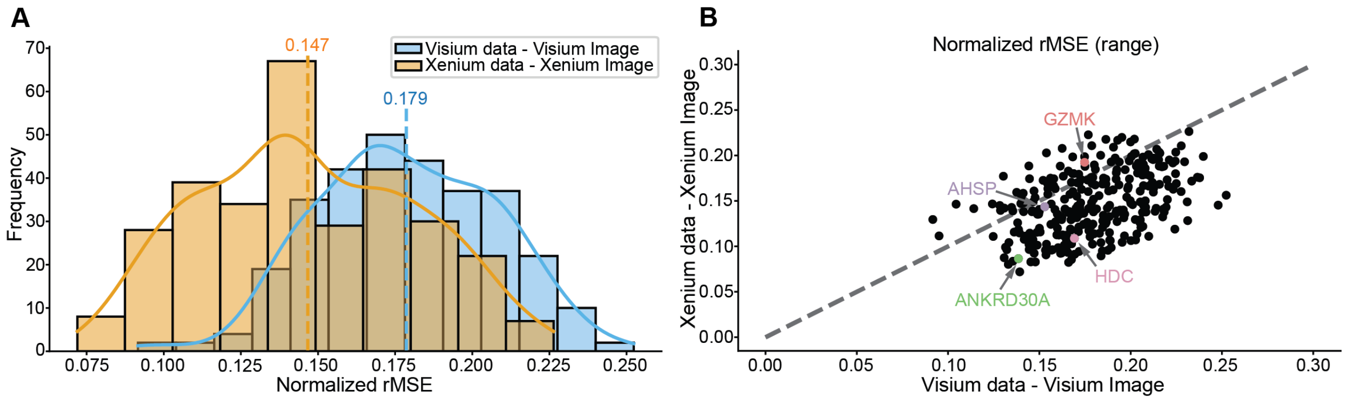

Supplementary Figure 2: Prediction performance using normalized rMSE for Visium vs. Xenium data. A. Histogram showing the distribution of normalized rMSE for gene expression predictions using Visium and Xenium data. The dotted vertical line denotes the mean rMSE, and the solid curved line traces the density estimate. Results are computed on the test set and represent the average performance across five independently trained models. **B.** Scatterplot comparing the normalized rMSE of predictions from Visium and Xenium data, based on the test set and averaged over five models. The gray dotted line denotes x=y.

bioRxiv preprint doi: https://doi.org/10.1101/2025.09.04.674228; this version posted September 9, 2025. The copyright holder for this preprint (which was not certified by peer review) is the author/funder, who has granted bioRxiv a license to display the preprint in perpetuity. It is made available under aCC-BY 4.0 International license.

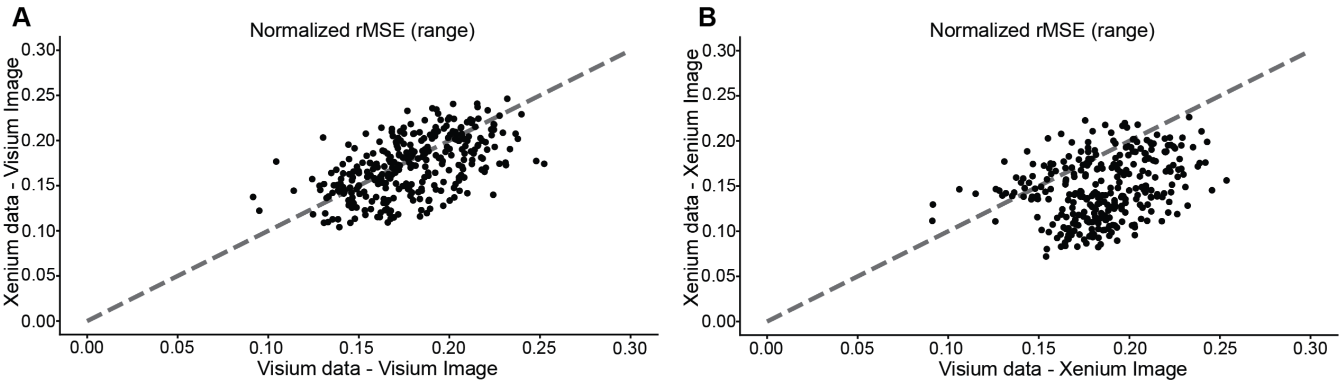

Supplementary Figure 3: Prediction performance using normalized rMSE for Visium vs. Xenium molecular data. Scatterplots of normalized rMSE for models trained on varied molecular inputs, evaluated on the held-out test set and averaged across five independent runs, using (A) the Visium histology image and (B) the Xenium histology image. The gray dotted line denotes x=y.

bioRxiv preprint doi: https://doi.org/10.1101/2025.09.04.674228; this version posted September 9, 2025. The copyright holder for this preprint (which was not certified by peer review) is the author/funder, who has granted bioRxiv a license to display the preprint in perpetuity. It is made available under aCC-BY 4.0 International license.

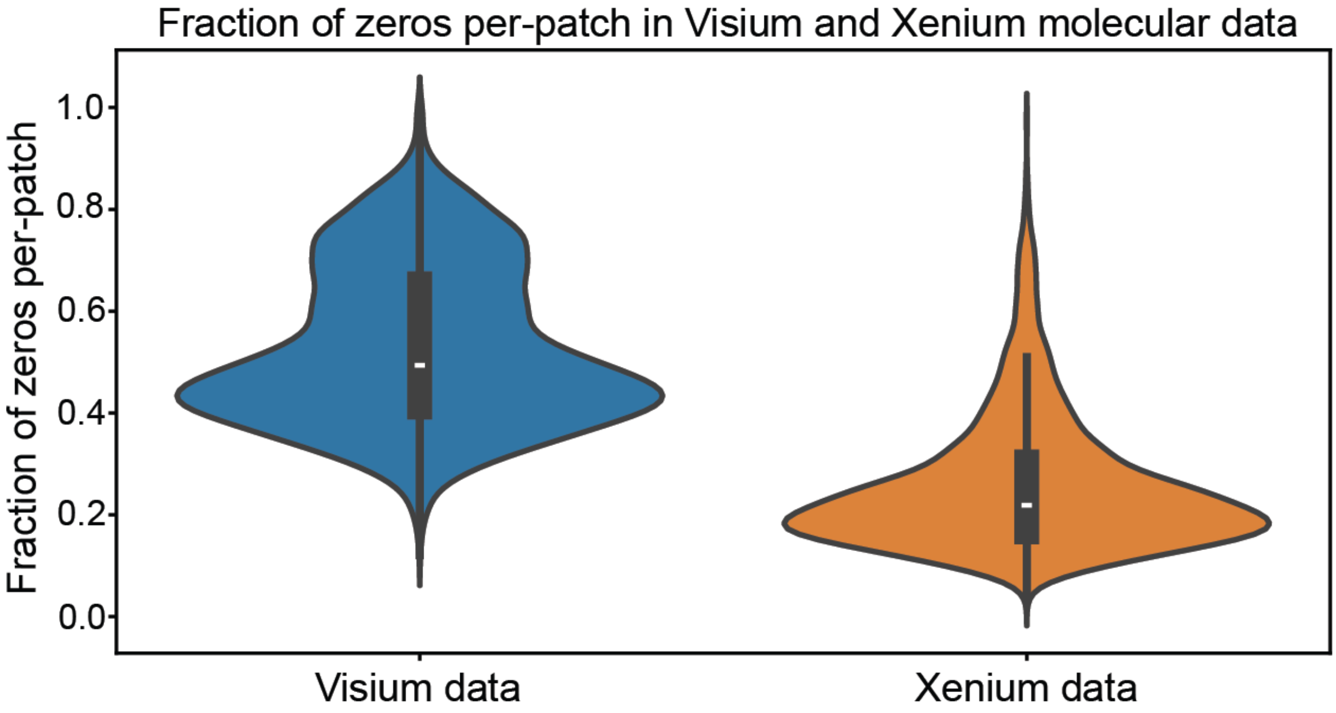

Supplementary Figure 4: Sparsity in Visium and Xenium molecular data. Violin plots of the per-patch fraction of zero counts in Visium and Xenium molecular data. The shape of each violin reflects the density of values along the y-axis, and the overlaid boxplot indicates the median and the 25th and 75th percentiles.

bioRxiv preprint doi: https://doi.org/10.1101/2025.09.04.674228; this version posted September 9, 2025. The copyright holder for this preprint (which was not certified by peer review) is the author/funder, who has granted bioRxiv a license to display the preprint in perpetuity. It is made available under aCC-BY 4.0 International license.

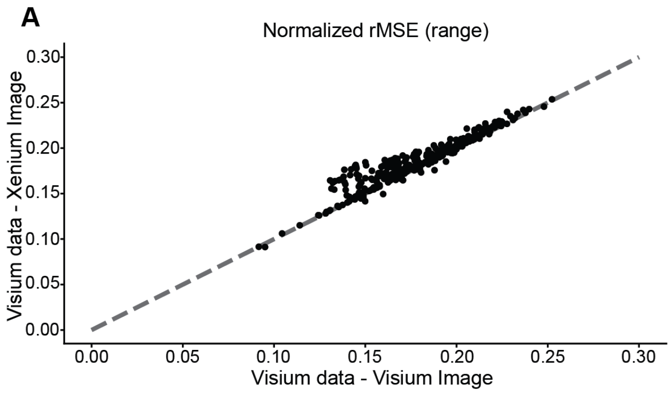

Supplementary Figure 5: Prediction performance using normalized rMSE for Visium vs. Xenium imaging data. Scatterplots of normalized RMSE for models trained on varied image inputs, evaluated on the held-out test set and averaged across five independent runs, using (A) the Visium molecular data and (B) the Xenium molecular data. The gray dotted line denotes x=y.

bioRxiv preprint doi: https://doi.org/10.1101/2025.09.04.674228; this version posted September 9, 2025. The copyright holder for this preprint (which was not certified by peer review) is the author/funder, who has granted bioRxiv a license to display the preprint in perpetuity. It is made available under aCC-BY 4.0 International license.

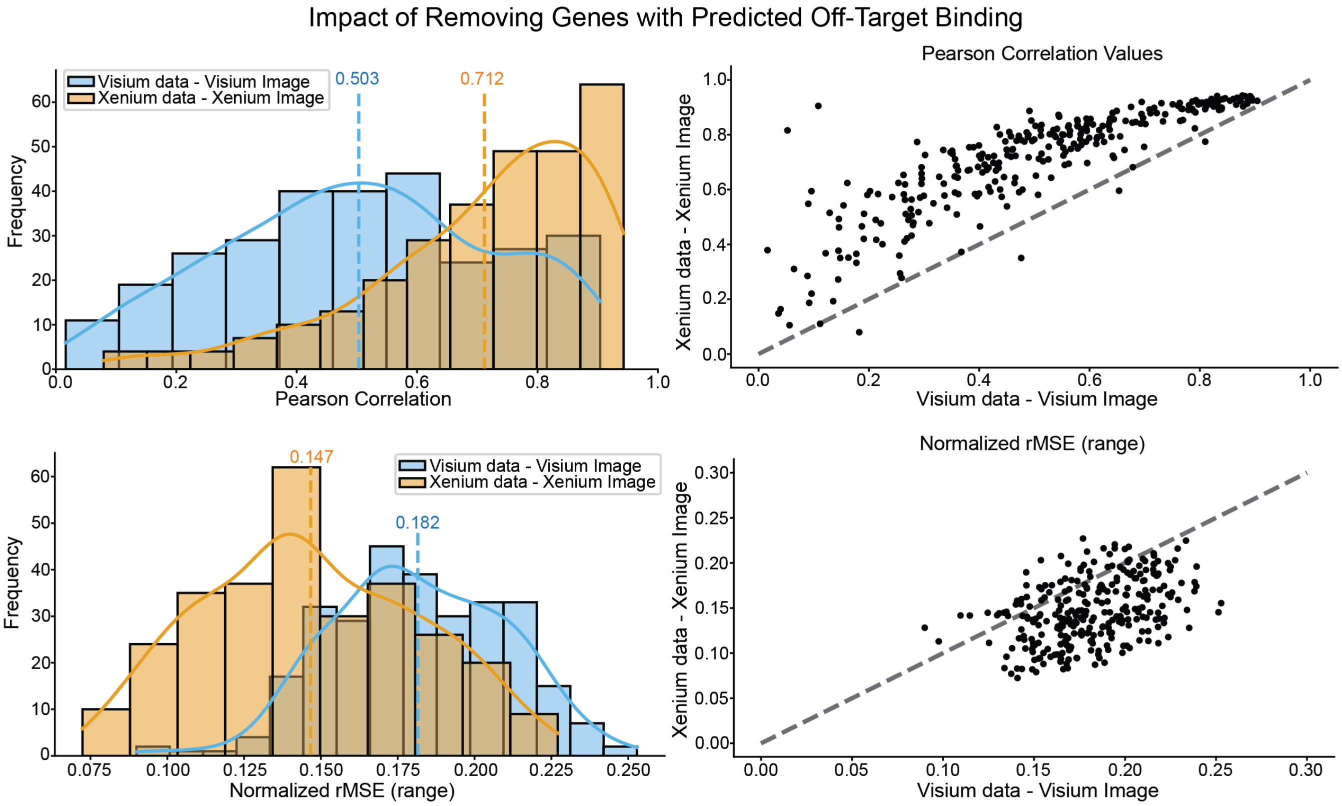

Supplementary Figure 6: Visium vs. Xenium results when removing genes with predicted off-target binding. A. Histogram showing the distribution of Pearson correlation for gene expression predictions using Visium and Xenium data. The dotted vertical line denotes the mean rMSE, and the solid curved line traces the density estimate. Results are computed on the test set and represent the average performance across five independently trained models. **B.** Scatterplot comparing the Pearson correlation of predictions from Visium and Xenium data, based on the test set and averaged over five models. The gray dotted line denotes x=y. **C.** Histogram showing the distribution of normalized rMSE for gene expression predictions using Visium and Xenium data. The dotted vertical line denotes the mean rMSE, and the solid curved line traces the density estimate. Results are computed on the test set and represent the average performance across five independently trained models. **B.** Scatterplot comparing the normalized rMSE of predictions from Visium and Xenium data, based on the test set and averaged over five models. The gray dotted line denotes x=y.

bioRxiv preprint doi: https://doi.org/10.1101/2025.09.04.674228; this version posted September 9, 2025. The copyright holder for this preprint (which was not certified by peer review) is the author/funder, who has granted bioRxiv a license to display the preprint in perpetuity. It is made available under aCC-BY 4.0 International license.

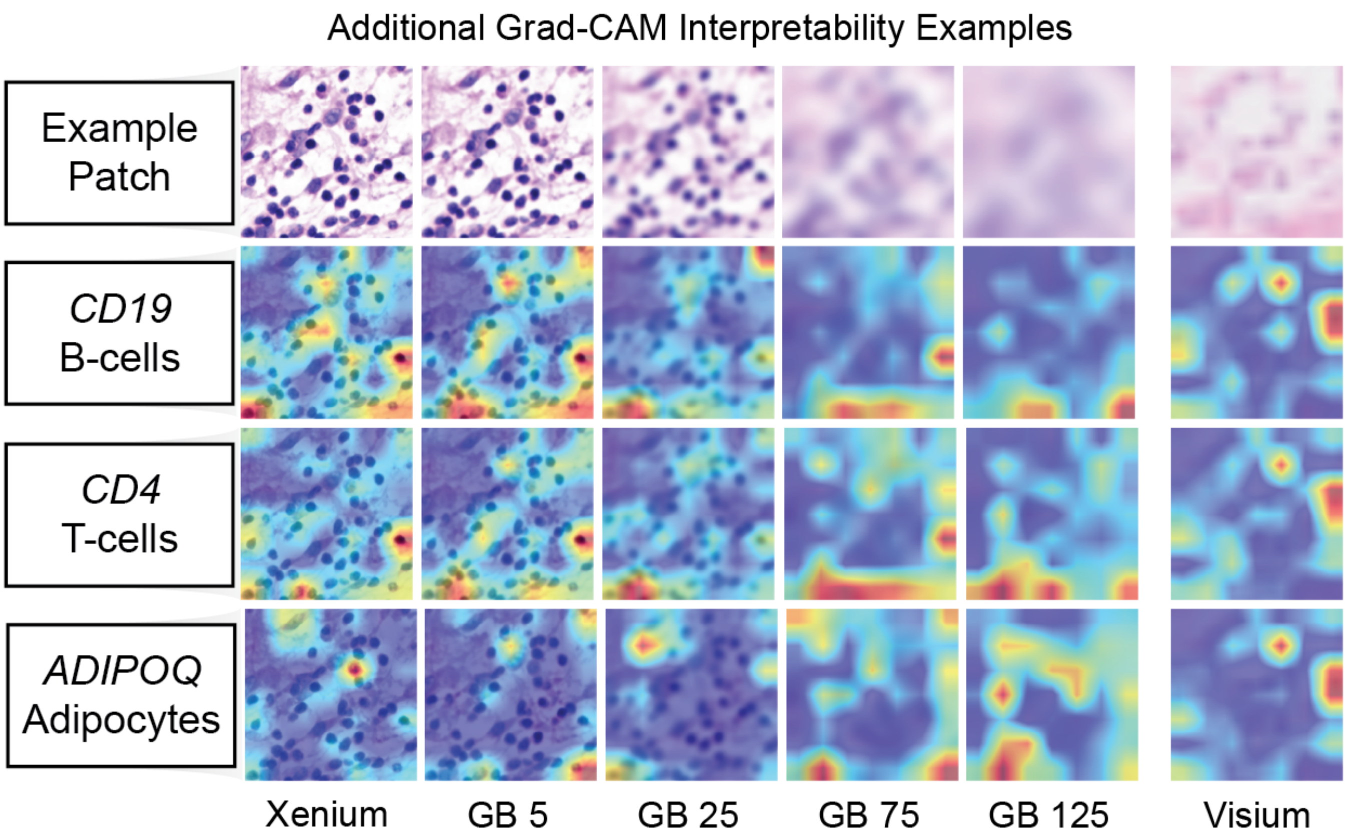

Supplementary Figure 7: Additional Grad-CAM interpretability examples. A representative histology patch is shown under increasing Gaussian blur (GB) levels, with corresponding Grad-CAM heatmaps for *CD19* (B-cell marker), *CD4* (T-cell marker), and *ADIPOQ* (adipocyte marker).
